# Do network synergies facilitates the realization of M&A motivation?: From the perspective of network node degree and strength change

**DOI:** 10.1371/journal.pone.0284204

**Published:** 2023-04-20

**Authors:** Ziqiao Zhang, Qiusheng Zhang, Chaofeng Li

**Affiliations:** 1 School of Accounting, Luoyang Institute of Science and Technology, Luoyang, China; 2 School of Economics and Management, Beijing Jiaotong University, Beijing, China; 3 China North Industries Corporation, Beijing, China; Alexandru Ioan Cuza University: Universitatea Alexandru Ioan Cuza, ROMANIA

## Abstract

The current evaluation of M&A performance lacks consideration of M&A motives. In this paper, we theoretically analyse and empirically test the effect of network synergy generated by M&A on the degree of realization of corporate M&A motives and the mechanism of its effect by constructing an equity network between a listed company and its subsidiaries within the company. The results show that the greater the variation of internal network node degree and strength, the more beneficial it is to promote the degree of realization of corporate M&A motivation; the results of further mechanism of action tests show that the variation of network node degree and strength has significant effects on economies of scale, economies of scope, and transaction costs; Furthermore, the heterogeneity test finds that the effect of variation of network node degree and strength to promote the degree of realization of corporate M&A motivation is more significant in the case of non-cash payment method and related M&A. This paper extends the study of complex networks to the field of M&A and uniquely explains the paradox of the "high failure rate" of M&A and the increasing activity of M&A activities from the perspective of network synergy, which helps to rationalize the M&A behavior of enterprises and further regulate the M&A behavior of listed companies by regulatory authorities.

## 1 Introduction

Known as one of the most important strategic investment methods for enterprises to use the capital market to improve quality and efficiency, gain network synergies, and cultivate competitive advantages in the market, mergers and acquisitions (M&A) have had a significant impact on the development of a modern industrial system and on the high-quality development of the Chinese economy in recent years. According to the "Review of China’s M&A market in 2020 and prospect in 2021’ released by PwC", the deal value of M&A activities in China increased by 30% in 2020 to reach $7,338 billion US dollars, the highest level since 2016. However, scholarly research indicates that the growing M&A activity is accompanied by a high rate of failure [1]. M&A activity frequently fails to generate value for shareholders and, in some cases, can detract from the value of the firm [2, 3]. This paradox has become a scientific issue at the forefront of M&A [4].

The mainstream approach to evaluate M&A performance is to compare cumulative abnormal stock returns, long-term shareholder value, and changes in financial metrics before and after the acquisition. However, these approaches may overlook the "original intent" of M&A motives and result in inaccurate conclusions. Several Scholars attempted to evaluate M&A performance from the perspective of M&A motives, and Brouthers et al. [5] further argued that the success and failure of M&A should be measured by examining the extent to which M&A motives are realized. In existing studies, some researchers have made different classifications of M&A motives, however, they just used single indicators or synthetic indicators obtained through factor analysis and principal component analysis instead of combining all types of motives to evaluate the performance [6, 7]. Even though some researchers have attempted to evaluate M&A performance through different types of M&A motives, they have only constructed evaluation systems theoretically without thorough testing [8, 9]. Most of the M&A performance information from existing tests was obtained either through questionnaires asking executives to rate relevant indicators, asking reviewers to comment on relevant indicators, or using hierarchical analysis, which are all empowerment methods that assign weights to relevant indicators [10, 11]. However, these approaches cannot accurately reflect the M&A performance, and there is currently no empirical test for large samples or research results on the mechanisms that influence the degree of realization of corporate M&A motivation.

Under the background of the new era, enterprise networking features become increasingly distinct, and competition among businesses have transformed into competition among businesses’ economic networks. The enterprise economic network is a complex system jointly formed by an enterprise’s internal economic relationship based on control relationships and inter-firm economic networks embedded in enterprises. Since M&A is the merging and rearrangement of two economic systems, and the network economy can modify the structure of the enterprise economic network before and after the merger to build a network economy, the network economy becomes the most important source of synergy effects of M&A in the enterprise economic network. However, in recent years, goodwill has been frequently “mined” by companies, including failures to meet performance expectations for high premium M&A and high failure rates for M&A. The reasons for these phenomena include, but are not limited to, insufficient understanding of the economics of corporate M&A and insufficient network economy theory and applied research to clearly guide the practice of business combinations. Therefore, it is a great theoretical and practical significance to investigate the influence of network synergies generated by changes in the acquirers’ economic network before and after mergers and acquisitions on the realization of M&A motivations.

The purpose of this paper is to examine whether network synergy has an impact on the degree of M&A motivation realization from the perspective of network node degree and strength change in M&A affairs occurring in China’s capital market. Its aims to provide theoretical and empirical evidence on the rationality of the degree of M&A motivation as a criterion for determining the success or failure of M&A. (2) To analyze and explain the relative contribution of network synergy on the degree of M&A motivation. (3) To guide M&A practice and provide useful theoretical support to further regulate corporate M&A behavior.

The remainder of this paper are organized as follows: Section 2 conducts theoretical analysis and develops our research hypothesis. Section 3 describes our empirical models. we present the date source, sample selection, and the main empirical results of the simulations and give some discussions in the fourth section. Finally, we show the relevant conclusions and the outlook of this work in the fifth section.

## 2 Methodology and hypothesis development

### 2.1 Economic networks and network synergies

For Chinese businesses operating in a transitional economy and a traditional relational society, the informal system of social networks is critical for corporate governance and resource allocation. The application of social networks in enterprises promotes the establishment and development of economic networks. Through interconnection, businesses build networks to acquire scarce resources such as knowledge and information through a collection of mutual relationships [12, 13]. The capacity of organizations to access network resources is determined by their positions in the network. Firms are driven to move in the network to find more resources in new and suitable places as part of the growth process, which promotes the evolution of economic networks in terms of structure and economic strength. According to Burt [14], network centrality and structural holes influence a firm’s position in the network. Firms with higher network centrality also have higher authority and reputation, and firms occupying structural hole positions tend to have rich, diverse, and differentiated resources such as knowledge and information. In addition, the more important the network position is, the higher the level of business credit financing of the firm [15]. With the development of science and technology as well as the intensification of competition, economic network relationships have become increasingly complex. Thus, the node degree and strength have become the main indicators to measure the features of complex networks. The node degree measures the position of nodes in the network, which reflects the ability of nodes to obtain resources in the network and the breadth of cooperation between subjects. Usually, the larger the node degree is, the stronger its "intermediary role" in connecting other nodes in the network and the more obvious its role in the whole network. And the strength reflects how closely the node cooperates with other nodes in the network, which reflects the depth of cooperation between subjects. Furthermore, the topology of economic networks affects the efficiency of resource flow between nodes. As the topology of the economic network changes, the degree and strength of nodes in the economic network will also change, and the non-uniform network characteristics of the economic network cause resources to flow to high-dimensional nodes, which promotes the establishment of new economic links between nodes and nodes to generate a network economy and improves the enterprise competitiveness through network synergy capabilities [16].

### 2.2 Network synergy and M&A motivation realization

Mergers and acquisitions generate network synergies by changing the topology of economic networks. The synergistic effect of M&A is considered to be the "1+1>2" effect of the matching relationship between the merging parties. In other words, the overall performance of the group of firms is better than the simple sum of the performance of the individual parts. To achieve synergies in a business acquisition, the acquirer must either be able to limit the threats of existing and potential competitors in input markets, production processes, or output markets; or it must be able to develop new markets or encroach on competitors’ markets, rendering competitors unable to respond [17]. M&A is the merging and reorganization of two corporate economic systems, and the development of M&A can result in changes in the corporate economic network’s structure both before and after the merger. By combining the nodes of the acquirer and the acquiree, the inter-organizational network can be significantly reshaped, allowing the acquirer to gain control of the acquiree’s nodes, resulting in a more favorable structure for the post-merger company, thus creating synergies [18]. The network synergy of M&A is represented in the closeness of nodes in the network and the efficiency of network organizations. Specifically, the closeness of nodes is reflected by comparing firms’ internal and external transaction costs in the form of equity strength and contractual strength. And the efficiency of network organizations is reflected by economies of scale, economies of scope, and the effectiveness of network operations brought by the resources of individual nodes in the network.

The network synergies generated by M&A can have a significant impact on the realization of M&A motives. In the context of the "new normal" of China’s economy, explicit M&A motivation is crucial for firms to acquire innovation capabilities, which directly affects the realization of M&A strategies [19]. Currently, a rising number of scholars tend to use the medium- to long-term test of M&A performance to determine whether M&A motives are realized. M&A implies the collapse or fusion of pre-merger nodes and the acquirer’s ability to legally inherit and control the acquiree’s relationships, all of which have a significant impact on the acquirer’s structural position, particularly if the acquirer is looking for ways to improve its position. According to Sun et al. [20], the network location centrality, network location structure hole, and network capacity are the primary parameters to determine the network power. The network synergy of M&A promotes the optimization of overall resource allocation efficiency by influencing the position of nodes in the network so that enterprises could receive economic benefits from cost reduction and specialized division of labor to reduce various uncertainties in daily business activities [21]. Following the M&A, both parties’ internal resources are reallocated according to corporate strategy, and both parties’ relationship networks are reshaped according to strategic needs to achieve a superior network position and generate value and benefits for the combined economic system. Specifically, on the one hand, the change in the enterprise’s position in the network generates a structural advantage in terms of the increasing efficiency of the enterprise’s information acquisition and resource allocation; On another hand, the enterprise’s overall structure of the external economic network changes, and the stability of the whole enterprise network is enhanced, which means that the risk resistance of enterprise is further improved, leading to improved post-merger business performance. Based on the discussion above, the first hypothesis of this paper is stated as follows:

**Hypothesis 1:** The network synergy generated by M&A can improve the performance of the firm.

The network synergy generated by M&A can produce a "resource effect" that will enhance an enterprise’s resource endowment and ability to utilize resources, generate economies of scale and scope, and reduce internal and external transaction costs. According to resource-based theory, a company will obtain more resources and capabilities if it has a unique network structure. The subjects in the nodes of the relationship network, which are also called "social knots" in the network, can use the linkage paths in the network to exchange and disseminate information. The closer to the center of the network, the more useful information can be accessed, and the more breadth and depth of information can be obtained [22]. Therefore, acquirers seek to collaborate with enterprises with a distinctive and potentially economically advantageous network structure in their local area, either as partners or subsidiaries [23]. Moreover, if there are highly similar resources between firms, they will have an opportunity to benefit from economies of scale [24]. More concretely, firms can share R&D, technologies, procurement, production, operations, marketing plans, distribution channels, unified management, and sales forces to achieve economies of scale [25]. Furthermore, the advantages of network organization are that M&A can encourage specialized division and collaboration of labor among different firms and coordinate and combine strengths and specialties while eliminating their disadvantages and deficiencies and increasing marginal rewards, which creates economies of scale for the network organization as a whole [26]. Thus, hypothesis 2–1 is:

**Hypothesis 2–1:** The network synergies generated by M&A can enhance economies of scale.

By establishing inter-firm connections, firms can break down information barriers and effectively help them gain access to a variety of complementary resources that are not available internally [27–29]. Complementary resources provide opportunities for firms to gain economies of scope, create synergies and develop new resources and corresponding skills [24]. Dong [30] argued that communication and interaction between nodes in a network are complex and various in form, containing both feedback of knowledge, information, material, and energy and sharing of technology, information, management, and experience, forming a beneficial situation of complementary resources. Thereby, the relationship between nodes in the network will be strengthened in the process of cooperation and interaction, as well as the ability of the network to create value will be improved. M&A can rapidly change the existing network topology structure, which allows companies to acquire and utilize key complementary resources outside the enterprise, and enables them to operate a variety of different but related products at the same time. The stronger the complementarity between the products of the two parties, the smaller the differences in the requirements for technical equipment, management, and the quality of the personnel involved. With the increasing diversification of product categories, companies can achieve economies of scope and synergy effects, leading to improved resource utilization efficiency and enhanced competitive advantages. Thus, hypothesis 2–2 is:

**Hypothesis 2–2:** The network economy generated by M&A can enhance the economy of scope.

According to institutional economics, enterprise networks, alongside market and bureaucracy, is one way of resource allocation. The enterprise network is comprised of nodal firms that are linked by complementary strengths and resource sharing [31–33]. Through the intimate interaction of nodes in an enterprise economic network, nodes can receive resources such as information and knowledge from the external environment [34]. In the fluctuating and complex network environment, the company uses the effectiveness of relationships in its position to cross the barriers of ineffective relationships, thereby establishing a "build-govern" network position to achieve innovative development [35–37]. Networks are self-organizing alternatives to markets, which help to improve the efficiency of dedicated resources, at the same time, the trust mechanisms in networks can also help to save transaction costs [38]. Specifically, M&A can significantly reshape inter-organizational networks and accomplish network synergy by changing the association between nodes in the network to accelerate the flow of information and reduce the accessing cost of resources within the organization. Thus, companies with strong ties in the economic network can rapidly acquire resources such as information and knowledge of other companies. The closer a company is to the center of the economic network, the more information transmission channels it will have, the faster the speed of obtaining resources, the stronger the bargaining power with partners, and the lower the transaction cost. Based on the statements above, hypothesis 2–3 is:

**Hypothesis 2–3:** The network synergies generated by M&A can reduce transaction costs.

## 3 Research design

### 3.1 Sample selection and data

This paper selects listed companies in Shanghai and Shenzhen A-shares that underwent mergers and acquisitions from 2007–2019 as the research sample, and the required data are obtained from the Guotaian database (CSMAR) and the Wind database (Wind). The selection criteria are as followed: (1) exclude M&A companies treated by ST, *ST, and PT; (2) exclude listed companies in the financial and insurance industries; (3) exclude samples of asset divestiture, asset replacement, debt restructuring, share repurchase and other types of restructuring; (4) retain the sample of M&A companies whose acquirers are listed companies and select only those with the M&A progress mark "completed "; (5) exclude samples with M&A amounts less than 1 million yuan; (6) for the same company with multiple M&As completed in the same year, retaining the first M&A event completed by the company in that year to reduce the interaction between different M&A events; (7) exclude samples with incomplete key data; (8) Winsorize the tails of all continuous variables at the 1% and 99% quantiles to avoid the adverse effects of some samples with extreme values on the empirical results. After the above screening, a final sample of 2201 M&A is obtained.

### 3.2 Model specification and key variables

The main regression test models are:

$$Realization=\alpha_{0}+\alpha_{1}\Delta N+\alpha_{2}Control+\sum Industry+\sum Year+\varepsilon$$ (1)

$$Realization=\alpha_{0}+\alpha_{1}\Delta s+\alpha_{2}Control+\sum Industry+\sum Year+\varepsilon$$ (2)

In the model, Realization is the dependent variable that measures the degree of realization of the M&A motives, and ΔROE is used to examine the change in performance of the acquirer after the M&A. The difference between the acquirer’s ROE value in the year following the completion of the M&A (the first and second years) and the acquirer’s ROE value in the year preceding the completion of the M&A is used to determine the degree of realization of the M&A motive.

The variation of node degree (N) and strength (S), referred to ΔN and ΔS, in the internal and external networks of listed companies are proxy variables for network synergy. The node degree (N) refers to the sum of the number of nodes connected to a node, which reflects the node’s ability to obtain resources in the network. In general, the higher the node degree is, the stronger the "intermediary role" of the node in connecting other nodes in the complex network.

The calculation of node degree is Ni = ∑_j∈k_ a_ij_. Therefore, the calculation of change in node degree ΔN before and after the merger is:

ΔN = (node degree of listed companies in the year of M&A completion—node degree of listed companies in the year before M&A completion) /nodal degree of listed companies in the year before M&A completion.

The node strength (S) refers to the sum of all edge weights connected to the nodes, reflecting the closeness of cooperation with other subjects, and is an important characteristic variable for establishing cooperation and communication between enterprises and other network subjects [39–41]. The strength of network relationships is directly related to the ability of subjects to obtain various types of heterogeneous resources such as technology and knowledge. In general, the higher the node degree, the higher the "status" of the node in the network.

The calculation of node strength is Si = ∑_j∈k_ w_ij_. Therefore, the calculation of change in node strength ΔS before and after the merger is:

ΔS = (node strength of listed companies in the year of M&A completion–node strength of listed companies in the year before M&A completion) /node strength of listed companies in the year before M&A completion

The reason for using proportional calculation is that the change in the acquirer’s position in the network depends on its initial position. For example, a decrease in node degree of 0.25 is more meaningful for a company with an initial node degree of 0.5 (50% change) than for a company with an initial node degree of 0.75 (33% change).

This paper constructs an equity network formed by a listed company and its subsidiaries, so the node degree is the number of the listed company’s subsidiaries and the node strength is the sum of the listed company’s shareholdings in subsidiaries.

Referring to the studies of Chen et al. [42], Liu et al. [43], and Pan et al. [44], the following variables are controlled in the regression model: firm size (Size), firm solvency (Lev), cash flow (OCF), the proportion of independent directors (Indep), board size (Board), dual position status (Dual), first largest shareholder (Top1), related transactions (Relevance), type of M&A target (Type), nature of ownership of the M&A party (SOE), industry (Industry) and year (Year). The definition of these control variables is shown in **Table 1**.

**Table 1:** Definition and interpretation of each variable.

	Variable Name	Variable Symbols	Variable Explanation
Dependent variables	Degree of realization of M&A motives	Realization	The difference between the acquirer’s ROE in the year after the completion of the acquisition (year 1 / year 2) and the ROE in the year before the completion of the acquisition
Independent variables	Variation of node degree	ΔN	(Nodal degree of listed companies in the year of M&A completion—Nodal degree of listed companies in the year before M&A completion)/Nodal degree of listed companies in the year before M&A completion
	Variation of node strength	ΔS	(Strength of listed companies in the year of M&A completion—Strength of listed companies in the year before M&A completion)/ Strength of listed companies in the year before M&A completion
Control variables	Company Size	Size	Logarithm of total assets at the end of the year
	Corporate solvency	Lev	Total liabilities / total assets
	Cash Flow	OCF	Net cash flow from operating activities/total assets
	Percentage of independent directors	Indep	Number of independent directors/number of directors
	Board Size	Board	Number of Board of Directors
	Dual job situation	Dual	The chairman and general manager both take1, otherwise take0
	Percentage of shareholding of the largest shareholder	Top1	Number of shares held by the company’s largest shareholder/total number of shares of the company
	Related Transactions	Relevance	The M&A related M&Athat takes the value of 1, otherwise it is 0
	Types of M&A subjects	Type	Equity subjects are taken1, non-equity subjects are taken 0
	Nature of property rights of mergers and acquisitions	SOE	The merging party is a state-owned holding company that takes the value of 1, otherwise, it is 0
	Industry	Industry	According to the industry sector in which the acquirer is in (2012 year CSRC industry classification standard)
	Annual	Year	Year of Corporate M&A

## 4 Empirical results

### 4.1 Descriptive statistics and correlation analysis

Panel A in **Table 2** illustrates the M&A transaction characteristics of the sample. following Panel A, cash payment M&As accounted for 63.83%, and non-cash payment M&As accounted for 36.17%, indicating that cash payment is the main payment method in the Chinese M&A market; related-party M&As accounted for 35.85%, and non-related M&As accounted for 64.15%. M&As of asset restructuring accounted for 28.62%, and M&As of non-major asset restructuring accounted for 71.38%; The proportion of equity bids in M&A targets reached 94.91%, and the proportion of non-equity bids was only 5.09%; among the types of M&As, horizontal mergers, and acquisitions accounted for 10.36%, vertical mergers and acquisitions accounted for 54.70%, accounting for more than half of the total sample, mixed mergers accounted for 29.53%, and other types of mergers accounted for 5.41%. Panel B illustrates the stock market and industry distribution of the sample. Based on **Table 2**, M&A in the main board market accounted for 39.48%, and M&A in the SME board and GEM board accounted for 60.52%; the manufacturing industry had the largest sample with 66.79%, M&A in the information transmission, software, and information technology service industry accounted for 8.91%, and M&A in the wholesale and retail industry accounted for 4.50%.

**Table 2:** Descriptive statistics of the sample.

Panel A: M&A transaction characteristics of the sample						
	M&A Payment Method		Form of M&A transaction		Whether major asset restructuring	
	Cash payment	Non-Cash Payments	Related Transactions	Unrelated Transactions	Major Asset Restructuring	Non-major asset restructuring
Number of samples	1405	796	789	1412	630	1571
Percentage (%)	63.83%	36.17%	35.85%	64.15%	28.62%	71.38%
	Subject Classification		M&A Type			
	Equity underlying	Non-Equity Underlying	Horizontal M&A	Vertical M&A	Hybrid M&A	Other
Number of samples	2089	112	228	1204	650	119
Percentage (%)	94.91%	5.09%	10.36%	54.70%	29.53%	5.41%
Panel B: Sample Stock Market and Industry Distribution						
	Stock Market		Industry			
	Mainboard	SMB and GEM	Manufacturing	Information transmission, software and information technology services	Wholesale and retail trade	Other
Number of samples	869	1332	1470	196	99	436
Percentage (%)	39.48%	60.52%	66.79%	8.91%	4.50%	19.81%

**Table 3** shows the descriptive statistics of the main variables in the model. The results show that in the equity network, the means of the degree of realization of M&A motivation in the year of M&A completion, one year after completion, and two years after completion are -0.0004, -0.013, and -0.037 respectively, which indicated that the M&A motives of the companies in the sample are generally not realized, and the M&A has not improved the performance level of the enterprises. The mean of the changes in the node degree of listed companies is 0.6890, indicating that the number of subsidiaries of listed companies after the completion of M&A is greater than the number of subsidiaries of listed companies before M&A. The mean of the changes in the strength of listed companies is 0.7190, indicating that the strength of control of listed companies over subsidiaries after the completion of M&A is greater than the strength of control of listed companies over subsidiaries before M&A.

**Table 3:** Descriptive statistics of the main variables.

	N	Mean	Variance	Minimum value	1/4 Median	Median	3/4 Median	Maximum value
Realization_t_	2201	-0.0004	0.0784	-0.3314	-0.0290	-0.0016	0.0243	0.3279
Realization_t+1_	2201	-0.0130	0.1230	-0.6680	-0.0400	-0.0020	0.0310	0.3600
Realization_t+2_	2201	-0.0370	0.1790	-1.0360	-0.0550	-0.0090	0.0310	0.3630
ΔN	2201	0.6888	1.1329	-0.4545	0.1190	0.3333	0.7778	7
ΔS	2201	0.7192	1.2625	-0.4986	0.1124	0.3329	0.7917	8
Size	2201	22.1280	1.0826	20.1370	21.3640	21.9780	22.7400	25.5390
Lev	2201	0.4062	0.1899	0.0632	0.2535	0.3945	0.5469	0.8424
OCF	2201	0.0406	0.0628	-0.1436	0.0036	0.0392	0.0775	0.2081
Indep	2201	0.3757	0.0545	0.3333	0.3333	0.3333	0.4286	0.5714
Board	2201	8.4607	1.5369	5	7	9	9	13
Dual	2201	0.3012	0.4589	0	0	0	1	1
Top1	2201	34.0950	14.8260	8.5400	22.3000	32.1900	43.6400	74.9600
Relevance	2201	0.3585	0.4797	0	0	0	1	1
Type	2201	0.9491	0.2198	0	1	1	1	1
SOE	2201	0.2622	0.4399	0	0	0	1	1

**Table 4** shows the correlation analysis of the main variables. Overall, there is no serious collinearity problem among the control variables.

**Table 4:** Correlation analysis.

	Size	Lev	OCF	Indep	Board	Dual	Top1	Relevance	Type	SOE
Size	1									
Lev	0.543***	1								
OCF	0.005	-0.103***	1							
Indep	-0.029	-0.019	-0.034	1						
Board	0.256***	0.142***	0.069***	-0.559***	1					
Dual	-0.189***	-0.120***	-0.019	0.079***	-0.160***	1				
Top1	0.138***	0.116***	0.147***	0.029	0.025	-0.035*	1			
Relevance	0.250***	0.167***	0.034	-0.042**	0.110***	-0.123***	0.128***	1		
Type	-0.046**	-0.017	-0.019	-0.001	-0.041*	0.026	-0.111***	-0.055***	1	
SOE	0.392***	0.291***	0.041*	-0.052**	0.276***	-0.247***	0.222***	0.283***	-0.121***	1

Note

* indicates *P* < 0.1

** indicates *P* < 0.05

*** indicates *P* < 0.01.

### 4.2 Main regression results

This section examines the effects of changes in the node degree and strength of the firm’s internal network on the degree of realization of the M&A motives. The regression results for the year in which the M&A was completed are shown in **Table 5**. Firstly, the effects of changes in node degree ΔN and changes in strength ΔS of listed companies on the degree of realization of corporate M&A motives are examined individually, shown in columns (1) and (5). Secondly, the effects of changes in node degree *ΔN* and changes in strength *Δ*S of listed companies on the degree of realization of corporate M&A motives are examined jointly with the control variables, shown in columns (2) and (6). Thirdly, the effects are examined again by controlling Industry and Year, shown in columns (3) and (7). The last one examines the effects of adopting cluster analysis on Industry, shown in columns (4) and (8). The results show that the change in node degree (*ΔN*) and the change in node strength (ΔS) have a significant positive effect on the degree of realization of corporate M&A motivation (Realization_t_) in the year in which the M&A is completed.

**Table 5:** Multivariate regression results of the variation of node degree and strength within listed companies and Realizationt.

	(1)	(2)	(3)	(4)	(5)	(6)	(7)	(8)
	Single	more	Control	Industry Clustering	Single	more	Control	Industry Clustering
ΔN	0.0050***	0.0052***	0.0052***	0.0052***				
	(3.40)	(3.49)	(3.47)	(4.00)				
ΔS					0.0050***	0.0051***	0.0048***	0.0048***
					(3.82)	(3.79)	(3.57)	(3.64)
Size		0.0012	-0.0001	-0.0001		0.0011	-0.0003	-0.0003
		(0.62)	(-0.07)	(-0.08)		(0.57)	(-0.13)	(-0.16)
Lev		0.0303***	0.0263**	0.0263**		0.0302***	0.0264**	0.0264**
		(2.87)	(2.34)	(2.17)		(2.86)	(2.34)	(2.19)
OCF		0.1194***	0.1268***	0.1268***		0.1181***	0.1255***	0.1255***
		(4.41)	(4.56)	(3.01)		(4.37)	(4.51)	(2.99)
Indep		0.0126	0.0170	0.0170		0.0137	0.0175	0.0175
		(0.34)	(0.45)	(0.47)		(0.37)	(0.47)	(0.50)
Board		-0.0008	-0.0003	-0.0003		-0.0008	-0.0002	-0.0002
		(-0.57)	(-0.19)	(-0.18)		(-0.54)	(-0.16)	(-0.15)
Dual		0.0033	0.0031	0.0031**		0.0032	0.0030	0.0030*
		(0.88)	(0.83)	(2.15)		(0.86)	(0.80)	(1.97)
Top1		-0.0000	0.0000	0.0000		-0.0000	0.0000	0.0000
		(-0.29)	(0.19)	(0.30)		(-0.32)	(0.18)	(0.27)
Relevance		0.0045	0.0038	0.0038		0.0044	0.0038	0.0038
		(1.22)	(1.03)	(0.81)		(1.18)	(1.03)	(0.81)
Type		0.0065	0.0023	0.0023		0.0066	0.0024	0.0024
		(0.84)	(0.30)	(0.39)		(0.87)	(0.31)	(0.40)
SOE		-0.0001	0.0040	0.0040*		-0.0002	0.0039	0.0039*
		(-0.03)	(0.85)	(1.94)		(-0.04)	(0.83)	(1.92)
Year			Control	Control			Control	Control
Industry			Control	Control			Control	Control
_cons	-0.0039**	-0.0536	-0.1243**	-0.1243*	-0.0040**	-0.0523	-0.1205**	-0.1205*
	(-1.98)	(-1.20)	(-2.49)	(-2.07)	(-2.11)	(-1.17)	(-2.42)	(-2.02)
R2	0.0052	0.0210	0.0465	0.0465	0.0066	0.0220	0.0468	0.0468
N	2201	2201	2201	2201	2201	2201	2201	2201

Note

*, **, *** contribute to significant levels of 10%, 5%, and 1% respectively.

The regression results for one year after the completion of M&A are presented in **Table 6**. The results show that after controlling for year and industry and clustering the industry, the change in node degree of listed companies (ΔN) has a significant positive effect on the degree of realization of corporate M&A motivation (Realizationt+1) one year after M&A completion. Under the univariate tests, the change in node strength (ΔS) of listed companies positively affects the degree of realization of corporate M&A motivation (Realizationt+1) one year after M&A completion, and it is statistically significant at the 10% level of significance. After controlling for year and industry and clustering the industries, the change in node strength (ΔS) of listed companies still has a positive effect on the degree of realization of corporate M&A motivation (Realizationt+1) one year after M&A completion, and it is also statistically significant at the 10% level of significance.

**Table 6:** Multivariate regression results of the variation of node degree and strength of listed companies with Realizationt+1.

	(1)	(2)	(3)	(4)	(5)	(6)	(7)	(8)
	Single	more	Control	Industry Clustering	Single	more	Control	Industry Clustering
ΔN	0.0034	0.0033	0.0036	0.0036**				
	(1.48)	(1.42)	(1.53)	(2.28)				
ΔS					0.0037*	0.0034	0.0029	0.0029*
					(1.77)	(1.61)	(1.41)	(1.82)
Size		-0.0009	-0.0015	-0.0015		-0.0009	-0.0016	-0.0016
		(-0.28)	(-0.44)	(-0.55)		(-0.30)	(-0.47)	(-0.59)
Lev		0.0030	-0.0059	-0.0059		0.0029	-0.0059	-0.0059
		(0.18)	(-0.34)	(-0.29)		(0.18)	(-0.33)	(-0.28)
OCF		0.1906***	0.1856***	0.1856***		0.1899***	0.1845***	0.1845***
		(4.48)	(4.26)	(4.12)		(4.47)	(4.24)	(4.10)
Indep		-0.0044	-0.0093	-0.0093		-0.0034	-0.0095	-0.0095
		(-0.07)	(-0.16)	(-0.33)		(-0.06)	(-0.16)	(-0.34)
Board		0.0023	0.0025	0.0025		0.0024	0.0026	0.0026
		(1.07)	(1.15)	(0.90)		(1.08)	(1.16)	(0.92)
Dual		0.0048	0.0052	0.0052		0.0047	0.0051	0.0051
		(0.81)	(0.89)	(1.58)		(0.80)	(0.87)	(1.53)
Top1		0.0001	0.0001	0.0001		0.0001	0.0001	0.0001
		(0.45)	(0.53)	(0.46)		(0.44)	(0.54)	(0.46)
Relevance		0.0101*	0.0085	0.0085***		0.0100*	0.0086	0.0086***
		(1.75)	(1.46)	(3.42)		(1.72)	(1.48)	(3.49)
Type		0.0011	-0.0040	-0.0040		0.0012	-0.0040	-0.0040
		(0.09)	(-0.34)	(-0.45)		(0.10)	(-0.33)	(-0.45)
SOE		0.0039	0.0075	0.0075		0.0040	0.0074	0.0074
		(0.56)	(1.03)	(1.35)		(0.57)	(1.01)	(1.33)
Year			Control	Control			Control	Control
Industry			Control	Control			Control	Control
_cons	-0.0151***	-0.0329	-0.0584	-0.0584	-0.0153***	-0.0323	-0.0553	-0.0553
	(-4.92)	(-0.47)	(-0.75)	(-0.78)	(-5.10)	(-0.46)	(-0.71)	(-0.74)
R2	0.0010	0.0148	0.0460	0.0460	0.0014	0.0151	0.0458	0.0458
N	2201	2201	2201	2201	2201	2201	2201	2201

Note

*, **, *** contribute to significant levels of 10%, 5%, and 1% respectively.

The regression results for 2 years after the completion of M&A is presented in **Table 7**. The results show that the change in the node degree of listed companies (ΔN) and the change in node strength (ΔS) have a negative but insignificant effect on the degree of realization of corporate M&A motivation (Realization_t+2_) two years after the completion of M&A. The results for the year of M&A, the year after 1 year, and the year after 2 years show that the effect of network economic synergy becomes less pronounced as the integration of M&A takes longer. Overall, combining the regression results of the year of M&A completion, one year after, and two years after, the internal network economy generated by M&A can improve M&A performance and promote the realization of corporate M&A motives, therefore, the results are consistent with the Hypothesis 1.

**Table 7:** Multivariate regression results of the variation of node degree and strength of listed companies with Realizationt+2.

	(1)	(2)	(3)	(4)	(5)	(6)	(7)	(8)
	Single	more	Control	Industry Clustering	Single	more	Control	Industry Clustering
ΔN	-0.0016	-0.0017	-0.0020	-0.0020				
	(-0.47)	(-0.50)	(-0.59)	(-0.53)				
ΔS					-0.0024	-0.0027	-0.0027	-0.0027
					(-0.79)	(-0.89)	(-0.88)	(-0.72)
Size		-0.0089**	-0.0064	-0.0064		-0.0089**	-0.0064	-0.0064
		(-1.99)	(-1.36)	(-1.21)		(-1.98)	(-1.35)	(-1.22)
Lev		0.0109	0.0017	0.0017		0.0108	0.0017	0.0017
		(0.45)	(0.07)	(0.06)		(0.45)	(0.06)	(0.06)
OCF		0.1701***	0.1756***	0.1756**		0.1699***	0.1755***	0.1755**
		(2.75)	(2.79)	(2.24)		(2.75)	(2.79)	(2.25)
Indep		0.0083	-0.0062	-0.0062		0.0060	-0.0080	-0.0080
		(0.10)	(-0.07)	(-0.11)		(0.07)	(-0.09)	(-0.14)
Board		0.0007	-0.0004	-0.0004		0.0006	-0.0005	-0.0005
		(0.22)	(-0.13)	(-0.14)		(0.20)	(-0.14)	(-0.16)
Dual		-0.0104	-0.0079	-0.0079		-0.0104	-0.0079	-0.0079
		(-1.21)	(-0.93)	(-1.25)		(-1.21)	(-0.93)	(-1.25)
Top1		0.0007***	0.0006**	0.0006**		0.0007***	0.0006**	0.0006**
		(2.77)	(2.13)	(2.43)		(2.80)	(2.15)	(2.49)
Relevance		0.0038	0.0034	0.0034		0.0042	0.0037	0.0037
		(0.45)	(0.40)	(0.35)		(0.50)	(0.44)	(0.39)
Type		0.0069	0.0001	0.0001		0.0067	-0.0000	-0.0000
		(0.39)	(0.01)	(0.01)		(0.38)	(-0.00)	(-0.00)
SOE		0.0251**	0.0294***	0.0294**		0.0249**	0.0293***	0.0293**
		(2.47)	(2.79)	(2.46)		(2.45)	(2.78)	(2.47)
Year			Control	Control			Control	Control
Industry			Control	Control			Control	Control
_cons	-0.0358***	0.1045	0.0190	0.0190	-0.0352***	0.1059	0.0192	0.0192
	(-8.03)	(1.02)	(0.17)	(0.18)	(-8.03)	(1.04)	(0.17)	(0.18)
R2	0.0001	0.0154	0.0607	0.0607	0.0003	0.0156	0.0609	0.0609
N	2201	2201	2201	2201	2201	2201	2201	2201

Note

*, **, *** contribute to significant levels of 10%, 5%, and 1% respectively.

### 4.3 Heterogeneity tests

#### 4.3.1 Group test by M&A payment method

**Table 8** examines the impact of M&A payment methods on corporate M&A performance. The results show that there is a significant difference in the impact of network synergy on the degree of M&A motivation realization under non-cash payment and cash payment, with the use of non-cash payment method (mainly reflected in share-based payment M&A), the change in nodal degree (ΔN) and the change in node strength (ΔS) significantly contribute to the degree of realization of corporate M&A motivation and are statistically significant at the 1% level. It indicates that the original shareholders of the target company will become strategic investors, which makes the benefit bundling mechanism between the M&A parties stronger and the network economy has a greater impact on the degree of M&A motivation realization. When cash payment is used, the change in node degree (ΔN) and the change in strength (ΔS) have a negative but statistically insignificant effect on the degree of realization of corporate M&A motivation.

**Table 8:** Group test by M&A payment method.

	(1)	(2)	(3)	(4)	(5)	(6)	(7)	(8)
	Non-Cash	Cash	Non-Cash	Cash	Non-Cash	Cash	Non-Cash	Cash
ΔN	0.0072***	-0.0006	0.0069***	-0.0001				
	(3.52)	(-0.26)	(3.23)	(-0.06)				
ΔS					0.0072***	-0.0006	0.0067***	-0.0012
					(3.97)	(-0.28)	(3.57)	(-0.58)
Size			-0.0000	-0.0006			0.0001	-0.0006
			(-0.00)	(-0.23)			(0.02)	(-0.24)
Lev			0.0381*	0.0243*			0.0378*	0.0245*
			(1.75)	(1.84)			(1.74)	(1.85)
OCF			0.0979*	0.1379***			0.0959*	0.1379***
			(1.81)	(4.26)			(1.77)	(4.26)
Indep			0.0799	-0.0230			0.0846	-0.0232
			(1.24)	(-0.50)			(1.31)	(-0.50)
Board			-0.0003	-0.0004			-0.0002	-0.0003
			(-0.13)	(-0.20)			(-0.10)	(-0.19)
Dual			0.0058	0.0016			0.0059	0.0016
			(0.84)	(0.36)			(0.85)	(0.36)
Top1			0.0001	0.0000			0.0001	0.0000
			(0.29)	(0.08)			(0.23)	(0.10)
Relevance			0.0073	-0.0041			0.0067	-0.0042
			(1.11)	(-0.82)			(1.02)	(-0.85)
Type			0.0428***	-0.0100			0.0420***	-0.0101
			(2.68)	(-1.14)			(2.64)	(-1.16)
SOE			0.0120	-0.0008			0.0117	-0.0010
			(1.48)	(-0.14)			(1.44)	(-0.18)
Year			Control	Control			Control	Control
Industry			Control	Control			Control	Control
_cons	-0.0004	-0.0043*	-0.0873	-0.0618	-0.0010	-0.0043*	-0.0911	-0.0607
	(-0.11)	(-1.85)	(-0.67)	(-0.68)	(-0.28)	(-1.88)	(-0.70)	(-0.66)
R2	0.0153	0.0000	0.0751	0.0649	0.0194	0.0001	0.0778	0.0651
N	796	1405	796	1405	796	1405	796	1405

Note

*, **, *** contribute to significant levels of 10%, 5%, and 1% respectively.

#### 4.3.2 Group test by whether it is a related transaction

The related transaction also impacts the M&A activities significantly. As shown in **Table 9**, the internal network changes resulting from M&A in the sample of connected transactions significantly contribute to the degree of realization of corporate M&A motives, and the results are statistically significant. This indicates that there are fewer corporate cultural differences between the M&A subjects that have related transactions, which facilitates the smooth transfer of information between the acquiring parties and faster realization of M&A synergies to achieve the realization of M&A motives [45, 46]. However, M&A with unrelated transactions face a higher degree of information asymmetry, which makes it difficult to generate network synergies.

**Table 9:** Group test by whether they are related transactions.

	(1)	(2)	(3)	(4)	(5)	(6)	(7)	(8)
	Non-associated	Associations	Non-associated	Associations	Non-associated	Associations	Non-associated	Associations
ΔN	-0.0017	0.0095***	-0.0005	0.0098***				
	(-0.81)	(4.44)	(-0.22)	(4.46)				
ΔS					-0.0019	0.0094***	-0.0018	0.0096***
					(-1.00)	(4.99)	(-0.94)	(4.97)
Size			-0.0015	0.0002			-0.0016	0.0002
			(-0.58)	(0.06)			(-0.64)	(0.06)
Lev			0.0426***	-0.0005			0.0427***	0.0003
			(3.14)	(-0.03)			(3.15)	(0.01)
OCF			0.0939***	0.1708***			0.0939***	0.1690***
			(2.81)	(3.46)			(2.81)	(3.44)
Indep			-0.0108	0.0612			-0.0122	0.0623
			(-0.24)	(0.94)			(-0.27)	(0.96)
Board			0.0004	-0.0005			0.0004	-0.0006
			(0.26)	(-0.20)			(0.26)	(-0.23)
Dual			0.0057	-0.0006			0.0057	-0.0007
			(1.35)	(-0.08)			(1.36)	(-0.09)
Top1			-0.0000	0.0001			-0.0000	0.0001
			(-0.23)	(0.52)			(-0.19)	(0.48)
Type			0.0034	0.0048			0.0031	0.0044
			(0.34)	(0.38)			(0.32)	(0.35)
SOE			0.0003	0.0063			0.0001	0.0060
			(0.05)	(0.82)			(0.02)	(0.79)
Year			Control	Control			Control	Control
Industry			Control	Control			Control	Control
_cons	-0.0023	-0.0035	-0.0636	-0.0437	-0.0021	-0.0039	-0.0593	-0.0428
	(-0.99)	(-0.98)	(-0.69)	(-0.43)	(-0.93)	(-1.13)	(-0.64)	(-0.43)
R2	0.0005	0.0245	0.0721	0.0723	0.0007	0.0307	0.0727	0.0780
N	1412	789	1412	789	1412	789	1412	789

Note

*, **, *** contribute to significant levels of 10%, 5%, and 1% respectively.

### 4.4 Robustness test

#### 4.4.1 Changing the dependent variable

Under this test, the regressions are conducted using ΔROA and ROA in the year of M&A completion as substitute variables for the degree of realization of corporate M&A motives respectively, and the remaining variables are kept constant. The regression results are shown in **Appendix 1, 2 in S1 Appendix (**Robustness test**)**, and the results are still consistent with the hypothesis.

#### 4.4.2 Adding new control variables

The control variables of M&A transaction size and payment method are added to test the effects of changes in the node degree (ΔN) and changes in node strength (ΔS) of listed companies on the degree of realization of corporate M&A motives. The regression results are shown in **Appendix 3 in S1 Appendix (**Robustness test**)**, and the results still support the hypothesis.

## 5 Supplementary test

To test the impacts of network synergy effects on economies of scale, economies of scope, and transaction costs due to changes in economic network nodes, models (3)-(8) are constructed to test hypotheses H2-1 - H2-3:

$$Scale=\beta_{0}+a\Delta N+\beta Control+\sum Industry+\sum Year+\varepsilon_{1}$$ (3)

$$Scale=\beta_{0}+a\Delta S+\beta Control+\sum Industry+\sum Year+\varepsilon_{2}$$ (4)

$$Scpoe=\beta_{0}+a\Delta N+\beta Control+\sum Industry+\sum Year+\varepsilon_{3}$$ (5)

$$Scpoe=\beta_{0}+a\Delta S+\beta Control+\sum Industry+\sum Year+\varepsilon_{4}$$ (6)

$$Cost=\beta_{0}+a\Delta N+\beta Control+\sum Industry+\sum Year+\varepsilon_{5}$$ (7)

$$Cost=\beta_{0}+a\Delta S+\beta Control+\sum Industry+\sum Year+\varepsilon_{6}$$ (8)

### 5.1 Measurement of economies of scale

For the measure of economies of scale (Scale), referring to the study of Zhang and He [47], after M&A, the acquirer and the acquiree become one enterprise, and the two supplier relationship networks are synthesized into one supplier relationship network. Facing the same suppliers before, the acquirer has more voice after M&A, and the original suppliers are more willing to establish stronger cooperative relationships with the enterprise. The enterprise’s bargaining power over suppliers is enhanced, and its reliance on the top few suppliers is reduced, thus reducing procurement costs and effectively reducing the risk of market fluctuations. Therefore, the change in the ratio of the top five suppliers’ procurement amount to the company’s annual procurement amount before and after the M&A is used to measure the economy of scale, which is calculated as follows:

Scale = Proportion of the annual procurement amount of the top five suppliers in the year of completion of the merger and acquisition—Proportion of the annual procurement amount of the top five suppliers in the year before the completion of the merger and acquisition

If the result is positive, it indicates that the dependence of the company on the supplier increases after the completion of the M&A. If the result is negative, it indicates that the dependence of the company on the supplier decreases, and the scale effect increases. The greater the decrease in this value, the greater the scale effect.

### 5.2 Measurement of economies of scope

The economy of scope (Scope) in this paper is measured by using the revenue Herfindahl index and the revenue entropy index, which are widely used in the existing literature. The entropy index is calculated based on the proportion of revenue from each industry to total revenue based on the “Industry Classification Guidelines for Listed Companies revised by the China Securities Regulatory Commission” in 2012, the Wind database, and the segment reporting information disclosed in the annual reports of enterprises. The income Herfindahl index refers to the squared sum of the proportion of each industry’s income to the total income of the enterprise, and the larger this index is, the less diversified the enterprise is.

Therefore, this study uses the change in entropy index and Herfindahl index before and after the M&A, which is calculated as follows:

Scope1 = Entropy index in the year of M&A completion -Entropy index in the year before M&A completion

Scope2 = Herfindahl index in the year of M&A completion—Herfindahl index in the year before M&A completion

### 5.3 Measurement of transaction costs

For the measurement of transaction cost savings, Ang et al. [48] used the overhead cost indicator; and Xia and Liu [49] used the sum of selling expenses, administrative expenses, and financial expenses as a percentage of total assets to measure for a more comprehensive exploration of the components of transaction costs and to eliminate the interference of firm size. In this paper, regarding the above studies, the change in the sum of selling expenses, administrative expenses, and financial expenses as a percentage of total assets is used to measure transaction costs. The calculation is as follows:

Cost = Selling, administrative and financial expenses as a percentage of total assets in the year in which the acquisition is completed—Selling, administrative and financial expenses as a percentage of total assets in the year before the completion of the acquisition

The regression results of the effects of changes in node degree (ΔN) and changes in node strength (ΔS) on economies of scale, economies of scope, and transaction costs in the economic network of listed companies are shown in **Table 10**.

**Table 10:** Regression results of network economies and economies of scale, economies of scope, and transaction cost.

	(1)	(2)	(3)	(4)	(5)	(6)	(7)	(8)
	Scale	Scale	Scope1	Scope1	Scope2	Scope2	Cost	Cost
ΔN	-1.6672***		0.0330***		-0.0197***		-0.0009**	
	(-5.49)		(4.04)		(-4.20)		(-1.99)	
ΔS		-1.4959***		0.0207***		-0.0123***		0.0000
		(-5.48)		(3.00)		(-4.20)		(0.23)
Size	0.2402	0.2840	-0.0028	-0.0038	0.0015	0.0021	-0.0020*	-0.0021*
	(0.53)	(0.63)	(-0.30)	(-0.41)	(0.27)	(0.38)	(-1.90)	(-1.94)
Lev	4.1594*	4.2400*	-0.0160	-0.0158	0.0100	0.0099	0.0128**	0.0126**
	(1.76)	(1.79)	(-0.31)	(-0.31)	(0.34)	(0.34)	(2.27)	(2.24)
OCF	-0.9597	-0.6823	-0.0641	-0.0856	0.0254	0.0383	0.0301**	0.0302**
	(-0.16)	(-0.11)	(-0.52)	(-0.70)	(0.36)	(0.54)	(2.25)	(2.25)
Indep	-7.9010	-7.8325	0.0228	0.0091	-0.0082	0.0002	0.0165	0.0173
	(-0.98)	(-0.97)	(0.14)	(0.05)	(-0.08)	(0.00)	(0.86)	(0.90)
Board	-0.3720	-0.3823	-0.0022	-0.0023	0.0003	0.0004	0.0006	0.0007
	(-1.19)	(-1.22)	(-0.37)	(-0.37)	(0.09)	(0.10)	(0.90)	(0.96)
Dual	-0.0439	-0.0215	0.0242	0.0221	-0.0151	-0.0139	0.0007	0.0005
	(-0.06)	(-0.03)	(1.38)	(1.26)	(-1.50)	(-1.38)	(0.37)	(0.26)
Top1	-0.0070	-0.0066	0.0001	0.0001	-0.0001	-0.0001	0.0001*	0.0001*
	(-0.28)	(-0.26)	(0.10)	(0.19)	(-0.22)	(-0.31)	(1.72)	(1.74)
Relevance	0.3768	0.3841	0.0122	0.0163	-0.0035	-0.0060	-0.0049***	-0.0052***
	(0.50)	(0.51)	(0.74)	(0.99)	(-0.37)	(-0.63)	(-2.63)	(-2.80)
Type	2.5387	2.6062	0.0393	0.0395	-0.0191	-0.0192	-0.0031	-0.0035
	(1.45)	(1.49)	(1.12)	(1.12)	(-0.94)	(-0.95)	(-0.83)	(-0.93)
SOE	-1.8749*	-1.8548*	-0.0006	-0.0024	0.0031	0.0042	0.0012	0.0013
	(-1.90)	(-1.88)	(-0.03)	(-0.12)	(0.27)	(0.36)	(0.54)	(0.60)
Year	Control	Control	Control	Control	Control	Control	Control	Control
Industry	Control	Control	Control	Control	Control	Control	Control	Control
_cons	-1.5238	-2.6205	-0.0609	-0.0335	0.0397	0.0233	-0.0066	-0.0091
	(-0.12)	(-0.21)	(-0.18)	(-0.10)	(0.20)	(0.12)	(-0.23)	(-0.32)
R2	0.0411	0.0411	0.0507	0.0455	0.0505	0.0448	0.0685	0.0654
F	1.7976	1.7952	1.9158	1.7091	1.9077	1.6816	2.4456	2.3258
N	1503	1503	1364	1364	1364	1364	1200	1200

Note

*, **, *** contribute to significant levels of 10%, 5%, and 1% respectively.

Columns (1), (3), (5), and (7) show that the change in the node degree (ΔN) of listed companies significantly impacts the economies of scale, economies of scope, and transaction costs, where the change in the node degree (ΔN) of listed companies promotes economies of scale and scope and reduces transaction costs. On other hand, Columns (2), (4), (6), and (8) show that the change in node strength (ΔS) of listed companies promotes economies of scale and scope but has no significant effect on transaction costs.

## 6 Conclusion

This paper constructs an equity network between listed companies and their subsidiaries, theoretically, analyses, and empirically tests the impact of network synergy on the degree of realization of corporate M&A motives, and draws the following conclusions using M&A activities in Shanghai and Shenzhen A-shares as a research sample from 2007 to 2019. (1) Mergers and acquisitions, as a method of merging and reshaping two economic networks, can lead to changes in the degree and strength of internal and external network nodes of enterprises. Generally, the greater the changes, the greater the network synergy effect it generates, which is conducive to promoting the realization of enterprises’ M&A motives. In non-cash payment methods and related M&A, changes in the degree and strength of network nodes have more significant effects on the degree of realization of corporate M&A motives. The network synergy generated by M&A can significantly enhance M&A performance, generate economies of scale and scope, and save transaction costs.

This paper provides a novel explanation for the frontier issue of the paradox of M&A’s "high failure rate" and increasing activity. By organically linking M&A motives and M&A network synergies to explain M&A behavior and effects, it provides new evidence for established studies to judge M&A success or failure in terms of whether M&A motives are realized. Moreover, it enriches the existing research on factors influencing the realization degree of M&A motivation. Based on the changes in the nodal degree and strength of corporate economic networks, a complex systems approach is used to construct a model to examine the relative contribution of M&A synergies on the realization degree of M&A motivation, forming a useful addition to the existing literature.

The findings of this study helps enterprises planning to implement M&A strategies to clarify their M&A motives, make rational judgments on network synergies, scientifically carry out M&A valuation and avoid excessive M&A premiums. Meanwhile, it helps the relevant regulatory authorities to formulate policies for further regulating the M&A behavior and disclosing M&A-related information of listed companies. Additionally, the findings are conducive to the formation of a modern industrial system and the high-quality development of China’s economy as well. Furthermore, it will facilitate the formation of macroeconomic networks, including the Eurasian "connectivity strategy", and the proper implementation of M&A activities of group companies.

Due to the limitations of information disclosure and data acquisition, the economic network constructed in this paper is based on the formal relationship between enterprises. However, the informal relationship between enterprises may also play an important role in the strategic practice of enterprises. Future research can consider building an economic network based on the informal relationship between enterprises. In addition, for the sake of simplifying the model and highlighting the research focus, the relevant factors affecting enterprise network collaboration in the model may not be comprehensive enough. Subsequent research can consider the degree of realization of M&A motivation based on the impairment of M&A goodwill, and further, deepen the research on the intermediary effect of the impact mechanism.

## 7 Discussions

The increasingly active M&A activity is accompanied by the "high failure rate". This paradox has become one of the problems that has long puzzled the theoretical and practical fields [4]. Some scholars have suggested that one of the reasons may be caused by empirical research is using an inaccurate measure of performance [50, 51]. Up to now, accumulation of abnormal returns of stocks, long-term shareholder value and changes in financial indicators before and after implementation are commonly used to evaluate M&A performance. While a number of researchers have acknowledged the shortcomings of using financial ratios or stock value to measure merger performance, Brouthers [5] suggest a better measure of merger success or failure is the degree to which the merger achieves these predetermined objectives. Therefore, Some scholars have tried to evaluate M&A performance from the perspective of M&A motivation, but when measuring M&A performance, they did not combine various motives to evaluate, or use questionnaires, index evaluation or other empowerment methods to evaluate the M&A motivation system. It cannot truly reflect the performance of M&A activities. There are no research results clarify the complicated mechanism that affect the realization of M&A motivation. By combining the respective nodes of the acquirer and the target, M&A can significantly reshape the inter-organizational network, thus creating synergies which will affect the efficiency of resource allocation, and then affects the economic performance of enterprises [18].

Learn from the previous research, this study constructs an equity network to explain the realization of M&A motivation and the complicated mechanism that why the realization is affected based on the idea of network synergies. The results show that the greater the variation of internal network node degree and strength, the more beneficial it is to promote the degree of realization of corporate M&A motivation. Furthermore, the network synergy generated by M&A can significantly enhance M&A performance, generate economies of scale and scope, and save transaction costs.

Based on network collaboration, this paper uses the realization degree of achievement of M&A motivation as the criterion for determining M&A success or failure, which not only expands the research field of complex networks, but also reasonably and uniquely explains the paradox of "high failure rate" and increasingly active M&A activities from a new perspective. This makes up for the shortcomings of existing studies that commonly used M&A performance indicators and their measurement methods may be detached from the M&A motivation, resulting in biased judgment results. Furthermore, we adopt a large sample statistical analysis method to measure the relative contribution of M&A synergy to the degree of M&A motivation, which enriches the research results on the factors influencing M&A motivation.

As a basic theoretical research result, this study is conducive to guiding M&A behavior to be more rational, and also to guiding M&A application research. As stated in the article, although the paper has achieved certain theoretical contributions and practical implications, there are still limitations and shortcomings that need subsequent improvement.

## Supporting information

S1 Dataset (XLSX)

S1 Appendix (DOCX)
