# Supplementary material for: Do network synergies facilitates the realization of M&A motivation?: From the perspective of network node degree and strength change
Source: PLoS One. 2023 Apr 20;18(4):e0284204. doi: 10.1371/journal.pone.0284204 (PMC10118086; doi:10.1371/journal.pone.0284204)
Supplement: S1 Appendix — (DOCX) [file pone.0284204.s002.docx]

**Appendix (robustness test)**

**Appendix 1** Multivariate regression results of the variation of node degree and strength of listed companies and ΔROA

|  | (1) | (2) | (3) | (4) | (5) | (6) | (7) | (8) |
| --- | --- | --- | --- | --- | --- | --- | --- | --- |
| $\Delta$N | 0.0016** | 0.0016** | 0.0016** | 0.0016*** |  |  |  |  |
|  | (2.14) | (2.16) | (2.19) | (4.95) |  |  |  |  |
| $\Delta$S |  |  |  |  | 0.0019*** | 0.0018*** | 0.0017** | 0.0017*** |
|  |  |  |  |  | (2.78) | (2.66) | (2.46) | (4.82) |
| Size |  | 0.0006 | 0.0001 | 0.0001 |  | 0.0006 | 0.0000 | 0.0000 |
|  |  | (0.64) | (0.05) | (0.08) |  | (0.62) | (0.02) | (0.02) |
| Lev |  | 0.0067 | 0.0032 | 0.0032 |  | 0.0067 | 0.0032 | 0.0032 |
|  |  | (1.26) | (0.56) | (0.68) |  | (1.26) | (0.57) | (0.68) |
| OCF |  | 0.0802*** | 0.0871*** | 0.0871*** |  | 0.0799*** | 0.0868*** | 0.0868*** |
|  |  | (5.86) | (6.20) | (3.39) |  | (5.85) | (6.18) | (3.37) |
| Indep |  | 0.0104 | 0.0140 | 0.0140 |  | 0.0111 | 0.0144 | 0.0144 |
|  |  | (0.55) | (0.74) | (0.82) |  | (0.59) | (0.76) | (0.85) |
| Board |  | -0.0004 | -0.0001 | -0.0001 |  | -0.0003 | -0.0001 | -0.0001 |
|  |  | (-0.52) | (-0.12) | (-0.12) |  | (-0.49) | (-0.09) | (-0.09) |
| Dual |  | 0.0016 | 0.0016 | 0.0016* |  | 0.0016 | 0.0016 | 0.0016* |
|  |  | (0.83) | (0.85) | (1.93) |  | (0.82) | (0.84) | (1.84) |
| Top1 |  | -0.0001* | -0.0001 | -0.0001** |  | -0.0001* | -0.0001 | -0.0001** |
|  |  | (-1.71) | (-1.33) | (-2.22) |  | (-1.75) | (-1.35) | (-2.24) |
| Relevance |  | 0.0063*** | 0.0061*** | 0.0061*** |  | 0.0062*** | 0.0060*** | 0.0060*** |
|  |  | (3.37) | (3.25) | (3.77) |  | (3.31) | (3.22) | (3.77) |
| Type |  | 0.0041 | 0.0024 | 0.0024 |  | 0.0042 | 0.0024 | 0.0024 |
| SOE |  | 0.0018 | 0.0044* | 0.0044*** |  | 0.0019 | 0.0044* | 0.0044*** |
|  |  | (0.82) | (1.89) | (3.44) |  | (0.84) | (1.89) | (3.51) |
| Year |  |  | Control | Control |  |  | Control | Control |
| Industry |  |  | Control | Control |  |  | Control | Control |
| _cons | -0.0033*** | -0.0278 | -0.0589** | -0.0589* | -0.0035*** | -0.0278 | -0.0580** | -0.0580* |
|  | (-3.33) | (-1.23) | (-2.34) | (-2.07) | (-3.63) | (-1.23) | (-2.31) | (-2.03) |
| R2 | 0.0021 | 0.0271 | 0.0522 | 0.0522 | 0.0035 | 0.0282 | 0.0528 | 0.0528 |
| N | 2201 | 2201 | 2201 | 2201 | 2201 | 2201 | 2201 | 2201 |

Note: *, **, *** contribute to significant levels of 10%, 5%, and 1% respectively.

**Appendix 2** Multiple Regression Results of Changes in node degree and strength of Listed Companies and ROA in the Year of M&A Completion

|  | (1) | (2) | (3) | (4) | (5) | (6) | (7) | (8) |
| --- | --- | --- | --- | --- | --- | --- | --- | --- |
| $\Delta$N | 0.0017** | 0.0018*** | 0.0017*** | 0.0017*** |  |  |  |  |
|  | (2.32) | (2.88) | (2.77) | (3.71) |  |  |  |  |
| $\Delta$S |  |  |  |  | 0.0019*** | 0.0019*** | 0.0017*** | 0.0017*** |
|  |  |  |  |  | (2.90) | (3.33) | (3.07) | (3.11) |
| Size |  | 0.0037*** | 0.0048*** | 0.0048*** |  | 0.0037*** | 0.0048*** | 0.0048*** |
|  |  | (4.40) | (5.44) | (5.20) |  | (4.36) | (5.40) | (5.19) |
| Lev |  | -0.0632*** | -0.0674*** | -0.0674*** |  | -0.0632*** | -0.0674*** | -0.0674*** |
|  |  | (-13.91) | (-14.15) | (-16.49) |  | (-13.93) | (-14.15) | (-16.44) |
| OCF |  | 0.2038*** | 0.2111*** | 0.2111*** |  | 0.2034*** | 0.2107*** | 0.2107*** |
|  |  | (17.51) | (17.95) | (4.66) |  | (17.50) | (17.94) | (4.65) |
| Indep |  | -0.0054 | -0.0065 | -0.0065 |  | -0.0048 | -0.0060 | -0.0060 |
|  |  | (-0.34) | (-0.41) | (-0.70) |  | (-0.30) | (-0.38) | (-0.66) |
| Board |  | 0.0006 | 0.0004 | 0.0004 |  | 0.0007 | 0.0004 | 0.0004 |
|  |  | (1.06) | (0.72) | (0.96) |  | (1.09) | (0.75) | (1.00) |
| Dual |  | 0.0005 | 0.0009 | 0.0009 |  | 0.0005 | 0.0009 | 0.0009 |
|  |  | (0.31) | (0.58) | (0.94) |  | (0.29) | (0.56) | (0.88) |
| Top1 |  | 0.0004*** | 0.0003*** | 0.0003*** |  | 0.0004*** | 0.0003*** | 0.0003*** |
|  |  | (7.06) | (6.52) | (9.60) |  | (7.03) | (6.50) | (9.62) |
| Relevance |  | -0.0034** | -0.0027* | -0.0027** |  | -0.0035** | -0.0028* | -0.0028** |
|  |  | (-2.12) | (-1.75) | (-2.74) |  | (-2.18) | (-1.78) | (-2.88) |
| Type |  | 0.0045 | 0.0067** | 0.0067 |  | 0.0046 | 0.0068** | 0.0068 |
|  |  | (1.38) | (2.06) | (1.54) |  | (1.40) | (2.08) | (1.53) |
| SOE |  | -0.0067*** | -0.0069*** | -0.0069*** |  | -0.0067*** | -0.0069*** | -0.0069*** |
|  |  | (-3.48) | (-3.49) | (-7.26) |  | (-3.48) | (-3.50) | (-7.31) |
| Year |  |  | Control | Control |  |  | Control | Control |
| Industry |  |  | Control | Control |  |  | Control | Control |
| _cons | 0.0436*** | -0.0382** | -0.0769*** | -0.0769*** | 0.0434*** | -0.0379** | -0.0759*** | -0.0759*** |
|  | (45.61) | (-1.99) | (-3.65) | (-5.44) | (46.20) | (-1.98) | (-3.61) | (-5.43) |
| R2 | 0.0024 | 0.2452 | 0.2891 | 0.2891 | 0.0038 | 0.2461 | 0.2897 | 0.2897 |
| N | 2201 | 2201 | 2201 | 2201 | 2201 | 2201 | 2201 | 2201 |

Note: *, **, *** contribute to significant levels of 10%, 5%, and 1% respectively.

**Appendix 3** Multiple regression results of the variation of node degree and strength of listed companies and Realizationt

|  | (1) | (2) | (3) | (4) | (5) | (6) | (7) | (8) |
| --- | --- | --- | --- | --- | --- | --- | --- | --- |
| $\Delta$N | 0.0050*** | 0.0041*** | 0.0041*** | 0.0041*** |  |  |  |  |
|  | (3.40) | (2.68) | (2.70) | (3.04) |  |  |  |  |
| $\Delta$S |  |  |  |  | 0.0050*** | 0.0041*** | 0.0038*** | 0.0038** |
|  |  |  |  |  | (3.82) | (2.99) | (2.78) | (2.77) |
| Size |  | -0.0011 | -0.0022 | -0.0022 |  | -0.0011 | -0.0023 | -0.0023 |
|  |  | (-0.49) | (-0.96) | (-0.95) |  | (-0.51) | (-1.01) | (-1.01) |
| Lev |  | 0.0348*** | 0.0304*** | 0.0304** |  | 0.0346*** | 0.0304*** | 0.0304** |
|  |  | (3.27) | (2.68) | (2.45) |  | (3.25) | (2.69) | (2.47) |
| OCF |  | 0.1209*** | 0.1298*** | 0.1298*** |  | 0.1197*** | 0.1286*** | 0.1286*** |
|  |  | (4.46) | (4.66) | (2.93) |  | (4.42) | (4.62) | (2.92) |
| Indep |  | 0.0089 | 0.0135 | 0.0135 |  | 0.0101 | 0.0141 | 0.0141 |
|  |  | (0.24) | (0.36) | (0.38) |  | (0.27) | (0.38) | (0.40) |
| Board |  | -0.0009 | -0.0005 | -0.0005 |  | -0.0009 | -0.0005 | -0.0005 |
|  |  | (-0.66) | (-0.35) | (-0.32) |  | (-0.63) | (-0.32) | (-0.30) |
| Dual |  | 0.0031 | 0.0029 | 0.0029* |  | 0.0031 | 0.0029 | 0.0029* |
|  |  | (0.83) | (0.78) | (2.06) |  | (0.81) | (0.76) | (1.93) |
| Top1 |  | -0.0000 | 0.0000 | 0.0000 |  | -0.0000 | 0.0000 | 0.0000 |
|  |  | (-0.03) | (0.33) | (0.51) |  | (-0.07) | (0.31) | (0.48) |
| Relevance |  | 0.0002 | -0.0003 | -0.0003 |  | 0.0002 | -0.0003 | -0.0003 |
|  |  | (0.05) | (-0.08) | (-0.06) |  | (0.05) | (-0.07) | (-0.05) |
| Type |  | 0.0059 | 0.0022 | 0.0022 |  | 0.0061 | 0.0023 | 0.0023 |
|  |  | (0.78) | (0.29) | (0.39) |  | (0.80) | (0.30) | (0.41) |
| SOE |  | 0.0005 | 0.0045 | 0.0045** |  | 0.0005 | 0.0044 | 0.0044* |
|  |  | (0.10) | (0.96) | (2.12) |  | (0.11) | (0.95) | (2.10) |
| Payment |  | -0.0053 | -0.0057 | -0.0057** |  | -0.0050 | -0.0054 | -0.0054** |
|  |  | (-1.18) | (-1.24) | (-2.35) |  | (-1.09) | (-1.18) | (-2.29) |
| Expense |  | 0.0023* | 0.0022* | 0.0022* |  | 0.0023* | 0.0023* | 0.0023* |
|  |  | (1.78) | (1.71) | (1.97) |  | (1.80) | (1.73) | (1.98) |
| Year |  |  | Control | Control |  |  | Control | Control |
| Industry |  |  | Control | Control |  |  | Control | Control |
| _cons | -0.0039** | -0.0413 | -0.1156** | -0.1156* | -0.0040** | -0.0413 | -0.1133** | -0.1133* |
|  | (-1.98) | (-0.90) | (-2.27) | (-2.01) | (-2.11) | (-0.90) | (-2.23) | (-1.98) |
| R2 | 0.0052 | 0.0253 | 0.0505 | 0.0505 | 0.0066 | 0.0261 | 0.0507 | 0.0507 |
| N | 2201 | 2201 | 2201 | 2201 | 2201 | 2201 | 2201 | 2201 |

Note: *, **, *** contribute to significant levels of 10%, 5%, and 1% respectively.
